# The Grand Challenge for Psychoanalysis and Neuropsychoanalysis: A Science of the Subject

**DOI:** 10.3389/fpsyg.2017.01259

**Published:** 2017-07-25

**Authors:** Ariane Bazan, Sandrine Detandt

**Affiliations:** Service de Psychologie Clinique et Différentielle, Faculté des Sciences Psychologiques et de l'Education, Centre de Recherche en Psychologie Clinique, Psychopathologie et Psychosomatique, Université Libre de Bruxelles Bruxelles, Belgium

**Keywords:** epistemology, repetition compulsion, neuropsychoanalysis, history of psychology, psychoanalysis, body mind, drive, mesolimbic dopamine

In 2011 we proposed that the modern advances in neurosciences would eventually push the field of psychology to an hour of truth as concerns its identity: indeed, what is psychology, if psychological functions and instances can be tied to characterized brain patterns (Bazan, 2011)? As Axel Cleeremans opens this Grand Challenge with a comparable question^1^, and as there is growing disagreement with the “I am my brain” paradigm, we think that the topic is indeed, 5 years later, crucially at stake. We had, in 2011, contextualized this question, as one driven by the advances in biology—anatomy in the sixteenth century, (neuro-)physiology in the nineteenth century and neurosciences today. Indeed, with each major advance, decisive moments came for psychology: in the sixteenth century, the name *psychologia* was launched, in the nineteenth century, psychology became a full-blown scientific field, and today, its specific identity is being questioned (Bazan, 2015). It now appears indeed that it is neuroscientists themselves, who formulate the possibility of a science of representational life, which is autonomous as regards to its biological substrates. For example, the neuroscientist Etienne Koechlin in a conference in Paris on February 2nd, 2016, gave as an alternative definition for neuroscience “the mechanisms and computational operations which govern the mental representations *independently from their material substrate and its content*^2^”. We will further propose that this autonomy is to be regarded as an organizational autonomy.

However, as psychology does not for now claim a clear identity of its own, it seeks refuge in the medical model when it comes to being practiced, i.e., as concerns clinical psychology, mainly. This has largely underestimated societal consequences. Indeed, the medical model is not adapted to the specificities of mental health (Bazan, 2013). Crucially, for example, the first intervention tool in mental health is the therapeutic alliance between patient and therapist (for review, see Wampold, 2001), and this is more important than the specific technique^3^, while in medicine the specific technical interventions are decisive for the treatment. We have proposed elsewhere (Bazan, 2013, 2015) how this medical model has a substantial counterproductive effect, creating, and sustaining mental health epidemics (mainly, by way of mediatized reification). The question of the specific identity of psychology thus has also societal and political implications—i.e., it is truly a grand challenge for our field.

In these 5 years of the section “Psychoanalysis and Neuropsychoanalysis” we have published a wide diversity of papers, which by their often outstanding quality can contribute to the dialogue of sciences. But, we'd venture to say, publishing in the field of psychoanalysis, turns out to be a real challenge. Indeed, if psychology might feel undecided as concerns its identity, this seems exponentially all the more true for psychoanalysis. Psychoanalysis, including in academia and in the dialogue with neuroscience, has the tendency to mellow some of its most controversial aspects. In our opinion, in as much as it indeed does so, it looses its interest for others and is cordially and gladly assimilated, not to say, neutralized. For example, in our opinion, psychoanalysis letting go of the drive concept, is a real loss for adjacent disciplines. Intuitively, we are all inclined to think that affect governs our behavior, for everything converges to show that we approach what is pleasurable, and avoid, fly away, from what is unpleasant. Thorndike (1898) made a law of that principle, Pavlov (1927) applied it, etc., and since then, we teach it to generations of students all over the world. Indeed, that someone would cling to something unpleasant, might be threatening to our idea of the human condition: but, the fact of the matter is, that the clinical practice shows just that; it is even one main reason for consultation. “Doctor, I know that it is bad for me, I am damaging myself, I'm about to loose my spouse and my job, but I just can't quit.” It is at the heart of addiction, of phobia, of obsession; it is at the heart of what we universally suffer from, namely, repetition compulsion.

The proposition of an organizational autonomy of the mental entails that there are no direct causal bridges between substrate and subjective experience, even if the biological substrate enables and constrains the mental dynamics governing the subjective experience. In other words, we would like to point a difference between the constraining role of the biological substrate—which is simultaneously its enabling role—for the advent of a mental organization, and its role in determining the meaning of mental experience, for which we posit mental constructs are more proximal, and therefore better, explanations. Repetition compulsion, now, is a good illustration of this difference between determination and constraint. A vertebrate body plan basically involves two bodies: an internal, invertebrate smooth muscle body constituted by the so-called vegetative bodily functions, where our needs originate (ex. air, food, liquid, defecation) and an external, vertebrate striated muscle body constituted by the skeleton and the skeletal muscles to move it. When a need arises in the internal body, it is the external body, which should realize in the world a so-called adequate act (Freud, 1905/1953:p. 184) to satisfy that need. As Freud (1905/1953:p. 184) says: “we may expect that Nature will have made safe provisions so that this experience of satisfaction shall not be left to chance.” Whenever a proper movement by surprise brings satisfaction, this is biologically marked by a dopamine peak at the level of the nucleus accumbens (Schultz, 1998), which will from that point on, enhance the probability of that action to be reactivated whenever stimuli are reminiscent of the inaugural event (see also, Bazan and Detandt, 2013). This dopamine incentive sensitization (Robinson and Berridge, 1993, 2000, 2003; Berridge, 2007) can be seen as a compensation mechanism for the fact that the external body arose at once with the emergence of vertebrates and took over the motor interaction of the organism with the external world. With organisms suddenly having two bodies with each their proper logic, there was need for a biological historicization system adjusting inner body needs to outer body actions. This is even more so the case in humans due to their premature condition when thrown into the world: humans much more than other animals are at loss on what adequate reaction could effectively discharge accumulating inner body tension, and, except for breathing, will depend on interactions with others for the organization of these actions. This means, that humans will biologically historicize their interactions with others. This is the basis for the repetition compulsion. For example, say that the first other is quite unreliable. Then, in order to maximize the chances for survival the child historicizes seduction techniques addressed to an unreliable other, and will have more probability in adult life to search for unreliable partners in order to re-deploy these seduction techniques. The bodily constraints—here, the need for a biological historicization mechanism for motor patterns deployed at inaugural moments—bring about a repetition compulsion in the interaction with others, which is constitutive for human mental life. Biology summons humans to the history of their (early) interactions, but does not dictate the mental experience of this repetition. In the experiential realm, properly mental logics for the organization of meaning take over, such as the tension between primary process associative tendencies (e.g., “this seemingly unreliable woman reminds me of my mother”) and secondary process reflexive reinterpretations (e.g., “however, as a grown-up now, I'll be smarter and deploy better strategies, or, alternatively, quit this endeavor altogether”). The clinical logic underlying the experience of the subject will be more readily grasped by these proximal mental dynamics, than what is feasible to infer from neuronal computations.

However, what might be an epistemological difference between psyche and soma? Let's again take the example of repetition compulsion. From a mental perspective, we have proposed that Lacan's theory^4^ deploys a logical order in which to situate the repetition compulsion (Bazan and Detandt, 2013). This temporal logic would involve (1) the emerging drive, (2) an experience of satisfaction, (3) accumulating bodily tension (upon reminiscing stimuli) and finally, (4) a repetition compulsion. Moreover, we have proposed that these four logical times found an equivalence in different aspects of the mesolimbic dopaminergic system, namely (1) Panksepp's seeking system (Panksepp, 1998); (2) Schultz's reward dopamine spike (Schultz, 1998); (3) Olds and Milner's “pleasure” center (Olds and Milner, 1954); and (4) Berridge and Robinson's incentive sensitization (*Ibid*.). Now, in a physiological framework these respective events do not seem to have a particular reciprocal order. Indeed, we propose that physiological processes are inherently cyclical: giving them a begin- or endpoint in time is always a matter of arbitrarily cutting a cycle. Order effects, at a biological level, seem unimportant—which is keenly illustrated by this famous question: what was first, the chicken or the egg? This is all the more true for neuronal processes, which, for the majority of them, proceed through cycling waves of neuron populations, and which, most of the time, are reciprocally connected. Therefore, our proposition is that the body has an extension in space, in such a way that it can be said of bodily elements that they are “left and right” (Snellius, 1594: p. 27^5^), but that the body is however not constitutively organized by time order effects. We propose that, in contrast, the mental apparatus has no constitutive extension in space but has an extension in time, in such a way that it can be specifically said of mental elements that they are “anterior or posterior to one another.” In other words, order effects are of constitutive importance for the mental, and therefore, in contrast with the cyclical nature of the physiological, are proposed to be inherently historical. In short, the mental reality is a dynamical reality, not a tissue reality.

This neuropsychoanalytic exercise concerning repetition compulsion has thus resulted in the proposition of a major epistemological difference between body and mind. Therefore, the relentless re-elaborating—through theory, clinical practice, and research—of the fundamental building blocks of metapsychology—such as e.g., primary and secondary processes, repression, repetition compulsion—in the current scientific context should be at the core of our concerns. The grand challenge, then, for psychoanalysis at this very moment in the history of sciences, in our opinion, is to go for its theory, and to stand by its counterintuitive, unpopular aspects, whatever the newest fashion reigning in the sciences of the mind, as long as these are both logically and clinically sound. We are privileged to hear subjects in their utmost intimacy, in things they often even don't share with their most loved ones. It is therefore our duty, if we are recipients of this kind of far reaching trust, to live up to this confidence, by giving the intimacy of what it means to be human, a voice in the larger scientific, and societal, debate.

To close this “grand challenge” we launch a call for new contributions in the large domain of psychoanalysis: we value original research, but also theoretical contributions, reviews or critical debates and research topics which pertain to psychoanalysis or neuropsychoanalysis, including epistemological perspectives upon the field. Let's take this opportunity with both hands to have this section of Frontiers function as a real, internationally valued tool for our field.

## Author contributions

All authors listed have made a substantial, direct and intellectual contribution to the work, and approved it for publication.

### Conflict of interest statement

The authors declare that the research was conducted in the absence of any commercial or financial relationships that could be construed as a potential conflict of interest. The handling Editor declared a shared affiliation, though no other collaboration, with the authors and states that the process nevertheless met the standards of a fair and objective review.

## Footnotes

^1^“It can rightfully be said that psychology lacks a clear identity”, http://journal.frontiersin.org/researchtopic/5440/grand-challenges-for-psychological-science-in-the-21st-century.

^2^Our translation of ≪ les mécanismes et les traitements computationnels qui régissent les représentations mentales *indépendamment de leur substrat matériel et de leur contenu* ≫. We propose that this alternative definition of neurosciences is, in fact, a definition of psychology.

^3^But see for a contradictory debate on this point, Keijsers (2014).

^4^This is his theory on *jouissance* (Lacan, 1986/1959–1960).

^5^“The physical things closer to natural bodies that move naturally, have an extension and for that reason occupy a space. (…) That is why geometrical properties are suited to natural bodies and, as different parts are in different places, they are in front, behind, above, below, left and right.” (Snellius, 1594, p. 26–27; our translation of the original Latin text).
